# Small molecule ERK5 kinase inhibitors paradoxically activate ERK5 signalling: be careful what you wish for…

**DOI:** 10.1042/BST20190338

**Published:** 2020-09-11

**Authors:** Simon J. Cook, Julie A. Tucker, Pamela A. Lochhead

**Affiliations:** 1 Signalling Laboratory, Babraham Institute, Babraham Research Campus, Cambridge CB22 3AT, U.K.; 2 York Biomedical Research Institute and Department of Biology, University of York, York YO10 5DD, U.K.

**Keywords:** extracellular signal-regulated kinases, kinases, paradoxical activation, small molecules

## Abstract

ERK5 is a protein kinase that also contains a nuclear localisation signal and a transcriptional transactivation domain. Inhibition of ERK5 has therapeutic potential in cancer and inflammation and this has prompted the development of ERK5 kinase inhibitors (ERK5i). However, few ERK5i programmes have taken account of the ERK5 transactivation domain. We have recently shown that the binding of small molecule ERK5i to the ERK5 kinase domain stimulates nuclear localisation and paradoxical activation of its transactivation domain. Other kinase inhibitors paradoxically activate their intended kinase target, in some cases leading to severe physiological consequences highlighting the importance of mitigating these effects. Here, we review the assays used to monitor ERK5 activities (kinase and transcriptional) in cells, the challenges faced in development of small molecule inhibitors to the ERK5 pathway, and classify the molecular mechanisms of paradoxical activation of protein kinases by kinase inhibitors.

## Introduction

Protein kinases play key roles in a variety of diseases including cancer and inflammation and have emerged as ‘druggable’ enzymes [1]. By 2019 some 48 small molecule protein kinase inhibitors had received USA FDA approval [2] with ∼175 more in clinical trials [3]. The principle of targeting protein kinases with small molecules is straightforward; block the active site so that it cannot bind ATP, prevent substrate binding, prevent binding of an up-stream activator or disrupt critical conformational changes. However, intensive research and clinical experience have identified two important limitations to the efficacy of kinase inhibitors. These are, innate or acquired resistance to the kinase inhibitor [4], and unintended activation of the target pathway, either by inhibition of negative feedback pathways [5] or through inhibitor binding to the kinase resulting in its paradoxical activation [6].

## The ERK5 signalling pathway in health and disease

**Figure 1.**
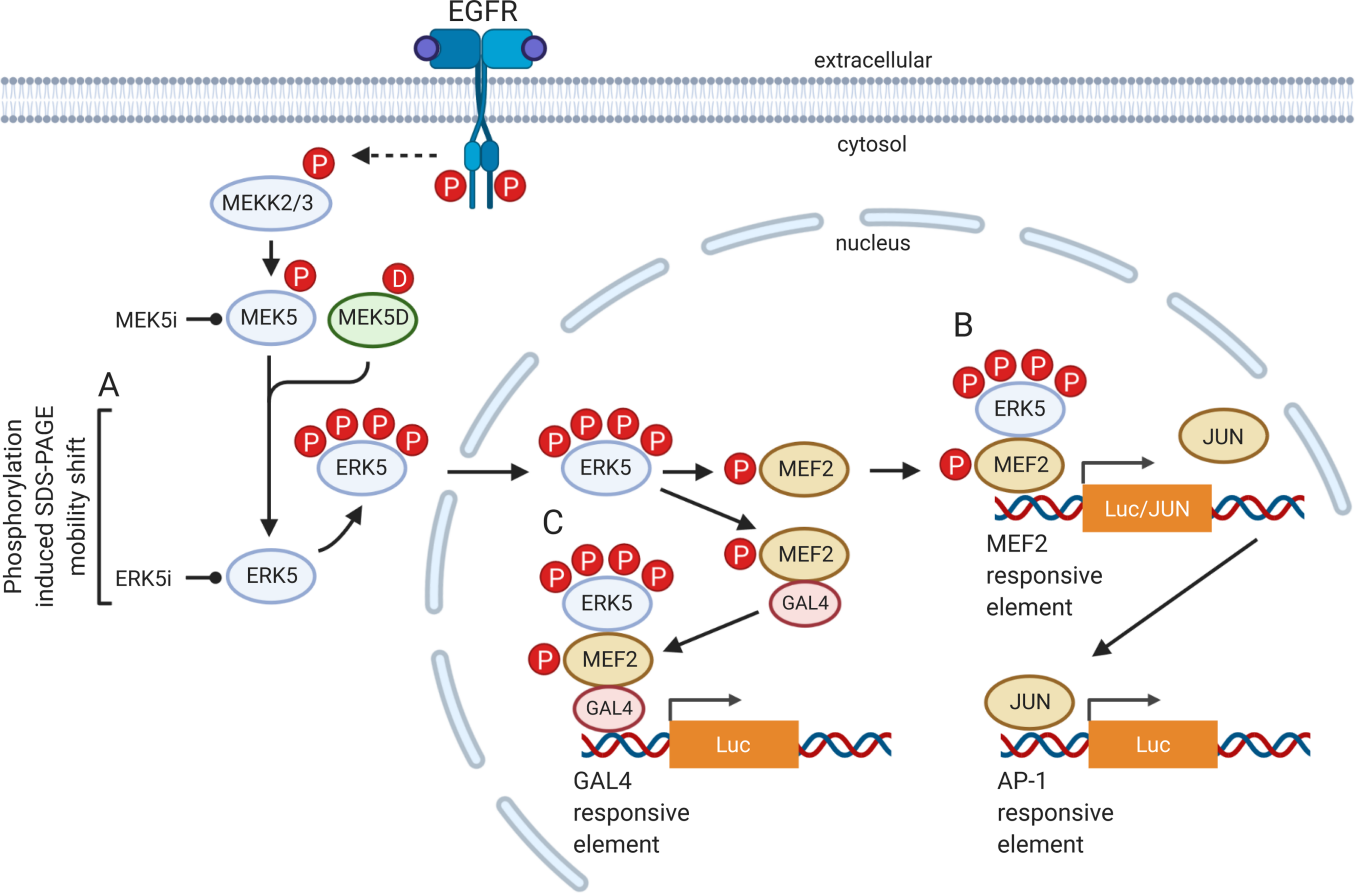
EGFR activation of the MEK5–ERK5 signalling pathway and the cell-based assays used to measure activation and inhibition by MEK5i and ERK5i (**A**) ERK5 autophosphorylation assay, (**B**) ERK5-driven MEF2 and AP-1 reporter assays, (**C**) ERK5-driven MEF2:GAL4 reporter assay. Figure created using bioRENDER.com.

**Figure 2.**
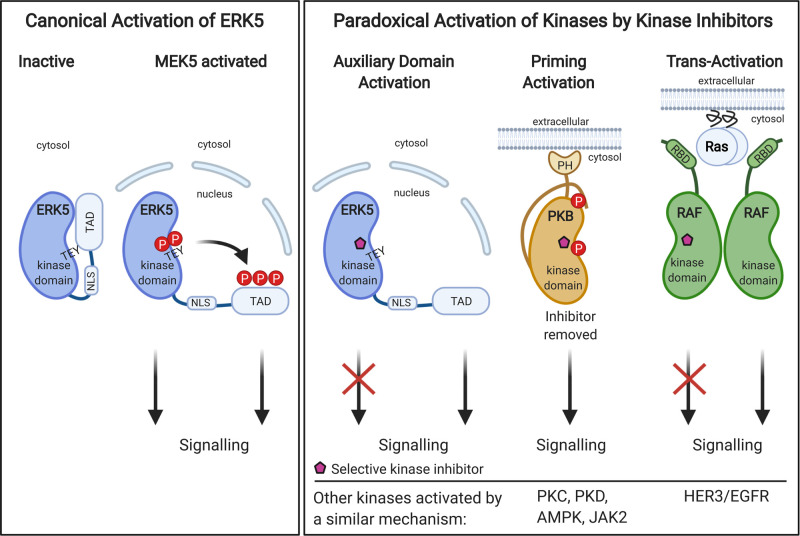
Canonical Activation of ERK5 and Paradoxical Activation of ERK5, PKB and RAF by kinase inhibitors. TAD, transactivation domain; NLS, nuclear localisation signal; PH, pleckstrin homology domain; RBD, RAS binding domain. Figure created using bioRENDER.com.

The mitogen-activated protein kinase (MAPK) family member, extracellular signal regulated kinase 5 (ERK5, also known as Big MAP Kinase 1 or BMK1) is encoded by the *MAPK7* gene [7,8]. It is the effector kinase of a three-tiered MAPK pathway, comprising MEKK2 and MEKK3 (the MKKKs), MEK5 (MKK) and finally ERK5 (MAPK) (Figure 1). ERK5 contains an N-terminal kinase domain that shares 50% identity with ERK2 [7,8] and a large, unique C-terminal extension that includes a nuclear localisation signal (NLS) and a transcriptional activation domain (TAD) [9] (Figure 2). Upon cellular stimulation (by mitogens [10], Toll-like receptor ligands [11] or cellular stresses [12]) activated MEK5 phosphorylates the TEY motif in the ERK5 activation loop leading to activation of its kinase domain [13], much like activation of ERK1/2 by MEK1/2. ERK5 then auto-phosphorylates its C-terminus which promotes ERK5 translocation from the cytosol to the nucleus [14,15] where ERK5 binds, phosphorylates and activates MEF2 transcription factors such as MEF2D [10,16,17] (Figures 1 and 2). The ERK5 C-terminus is also phosphorylated by other protein kinases including ERK1/2 [18], CDK1 [19,20] and CDK5 [21]. Thus, the C-terminus both mediates a subset of the effects of the ERK5 kinase domain and integrates signals from other pathways. ERK5 nuclear translocation and transcriptional activity is also regulated by CDC37-induced dissociation of Hsp90 [22], and SUMOylation [23]. For an excellent review on the mechanisms that regulate ERK5 nuclear localisation see Tubita et al*.* [24].

Components of the ERK5 pathway are ubiquitously expressed in adult tissues [7,8,14]. In development, *ERK5* regulates pluripotency in mouse embryonic stem cells [25], and *MEK5* and *ERK5* are required for blood vessel and cardiac development [26,27]. There is therapeutic potential in targeting the MEK5–ERK5 pathway in disease, especially cancer and inflammation. In this section, we have only considered data from knockdown and knockout experiments when assigning the therapeutic potential of inhibition of the ERK5 pathway. This is due to the pitfalls uncovered when generating ERK5i as therapeutics or tool compounds: off-target effects on kinases and bromo-domain containing proteins, and paradoxical activation (Table 1). We discuss these pitfalls in more detail later in the review. Knockdown of ERK5 or MEK5 by siRNA has shown anti-inflammatory effects in endothelial cells and monocytes [11,28]. Knockout of *ERK5* in tumour-associated macrophages impedes the growth of melanoma and lung carcinoma in mouse models [29]. Furthermore, knockout of *ERK5* in keratinocytes prevents inflammation-driven tumorigenesis [30]. Knockdown of ERK5 (or MEK5) by siRNA has also shown the therapeutic potential of the ERK5 pathway in mutant BRAF-driven melanoma [31,32,33], mutant KRAS-driven pancreatic ductal adenocarcinoma (PDAC) [34], as well as prostate [35], breast [36] and bladder cancers [37]. There are conflicting results obtained with ERK5 siRNA in hepatocellular carcinoma (HCC) [38,39]. In colorectal cancer, siRNA to ERK5 shows that ERK5 is required for the regulation of cancer stem-like cell properties and tumour-sphere growth [40], but not for inhibition of tumour cell proliferation [38].

**Table 1.** ERK5 inhibitors

Inhibitor /PubChem CID	Class of ERK5 inhibitor	IC_50_: assay	Other targets IC_50_: assay	Mechanism of action	Kinase inhibitor type [67]	Paradoxical activator (% of MEK5D effect)	Activity in tumour model(s)	Phase of development	References
BIX02188 /135398492	MEK5	4.3 nM: *in vitro* kinase assay 0.82–1.15 µM: in cell ERK5:MEF2C	*In vitro* kinase assays for: ERK5: 810 nM CSF1R:280 nM LCK: 390 nM KIT: 550 nM Kinases with >70% inhibition at 3 µM: Src Kinases with >70% inhibition at 10 µM: ABL CSF1R(FMS) KIT LCK using the University of Dundee and Invitrogen kinase selectivity testing	ATP competitive	Type I^‡^	NR	NT	Preclinical	[58]
BIX02189/135659062	MEK5	1.5 nM: *in vitro* kinase assay 0.26–0.53 µM: in cell ERK5:MEF2C 49.5 nM: in cell ERK5:MEF2D	*In vitro* kinase assays for: ERK5: 810 nM CSF1R:28 0nM LCK: 390 nM KIT: 550 nM Kinases with >70% inhibition at 3 µM: Src Kinases with >70% inhibition at 10 µM: ABL CSF1R(FMS) FGFR1 LCK RSK2 + 4 using the University of Dundee and Invitrogen kinase selectivity testing In cells: TGFβ-type 1R: 5 µM using a SMAD reporter	ATP competitive	Type I^‡^	NR	Yes	Preclinical	[55,58,85]
SC-1-181	MEK5	10 µM decreases phospho-ERK5 by 59%	10 µM had no effect on phospho-ERK1/2 No other data available	Not ATP competitive	Type III^†^	NR	NT	Preclinical	[61,62]
XMD8-92/46843772	Dual ERK5/BRD4	190 nM: in cell lysate KiNativ 130 nM: in live cells KiNativ 364 nM: *in vitro* kinase assay 240 nM: In cell ERK5 band-shift	LRRK2 (*in vitro* kinase assay): 59 nM BRD4 (BROMOscan assay): 170 nM Kinases with >90% displacement (KiNativ): DCAMKL1, TNK4 and PLK4	ATP competitive	Type I	24% at 1µM	Yes	Preclinical	[63,64,83,86–89]
AX15836/122705989	ERK5	8 nM: in cell lysate KiNativ 9 nM: in live cells KiNativ 31 nM: in cell ΔTAD-ERK5:MEF2D	BRD4 BROMOscan assay: 3.6 µM No other kinases inhibited >70% at 1 µM by KiNativ	ATP competitive	Type I*	200% at 3 µM	NT	Preclinical	[55,64]
XMD17-109 (Cmpd26, ERK5-IN-1)/71604307	Dual ERK5/BRD4	162 nM: *in vitro* kinase assay 90 nM: in cell EGF band-shift 4.2 µM: in cell AP-1 reporter 163 nM: in cell FL-ERK5:MEF2D 90 nM: in cell ΔTAD-ERK5:MEF2D	*In vitro* LRRK2: 339 nM BRD4-1 Binding assay: 217 nM No other selectivity data published	ATP competitive	Type I*	40% at 1 µM	NT	Preclinical	[45,63] [55]
XMD17-26 (Cmpd 25)	Dual ERK5/BRD4*	80 nM:in cell EGF band-shift 82 nM: *in vitro* kinase assay 152 nM: in cell FL-ERK5:MEF2D 28 nM: in cell ΔTAD-ERK5:MEF2D	No selectivity data published	ATP competitive	Type I	50% at 1 µM	NT	Preclinical	[44,55]
JWG-045 (XMD10-78)	ERK5/LRRK2	98 nM: *in vitro* kinase assay	BRD4 AlphaScreen™: 11 µM LRRK2 *in vitro* kinase assay: 289 nM No other selectivity data published	ATP competitive	Type I*	NT	NT	Preclinical	[25,32,45]
JWG-071/131842089	ERK5/LRRK2	88 nM: *in vitro* kinase assay	BRD4 AlphaScreen™: 5.42 µM LRRK2 *in vitro* kinase assay: 109 nM Kinases with >70% inhibition at 1 µM by KINOMEscan™ DCAMKL1/2 LRRK2 LRRK2 (G2019S) PLK4	ATP competitive	Type I*	NT	NT	Preclinical	[45]
Compound 46	ERK5	820 nM: *in vitro* 3 µM: in cell FL-ERK5:MEF2D 1 µM: in cell ΔTAD-ERK5:MEF2D	No binding to BRD4 at 20 µM. Kinases with ≥ 90% inhibition at 10 µM : DCAMKL3 JAK1 SLK MAP3K15 TYK2 JAK2 MST2 DCAMKL1 by KINOMEscan™	ATP competitive	Type I	5% at 10 µM	Yes	Preclinical	[56]
BAY-885/134128280	ERK5	35 nM: *in vitro* kinase assay 120 nM: in cell MEF2 reporter assay	No other kinase inh >70% at 1 µM by Eurofins kinase panel No binding to BRD4 at >20 µM	ATP competitive	Type I*	20% at 1 µM	NT	Preclinical	[57]
ADTL-EI1712	Dual ERK1/2 ERK5	1 µM KINOMEscan % inhibition ERK1 94% ERK2 91% ERK5 87%	Kinases with >71% inhibition at 1 µM: KIT AXL KINOMEscan by Eurofins	ATP competitive^¶^	Type I^¶^	NT	Yes	Preclinical	[66]
Compound 3	ERK5	42 nM: *in vitro*	>20 other kinases inhibited at 10 µM using the ThermoFisher standard kinase panel Not tested against BRD4.	ATP competitive	Type I	NT	NT	Preclinical	[68]
Compound 5	ERK5	2.3 µM: *in vitro*	Kinases with >80% inh at 10 µM: CSF1R FLT4 GSK3α SYK TYK2 using the ThermoFisher standard kinase panel. Not tested against BRD4.	Allosteric and ATP competitive	Type IV	NT	NT	Preclinical	[68]
TG-02 (SB1317, Zotiraciclib)/16739650	Multi-kinase inhibitor including ERK5	43 nM: *in vitro*	*In vitro* kinase assays for: CDK9: 3 nM CDK5: 4 nM CDK2: 5 nM CDK3: 8 nM CDK1: 9 nM Lck: 11 nM TYK2: 14 nM Fyn: 15 nM JAK2: 19 nM FLT3: 19 nM FLT3 D835Y: 21 Fms: 27 nM TYRO3: 36 nM CDK7: 37 nM ERK5: 43 nM P38δ: 56 nM JAK1: 59 nM	ATP competitive^§^	Type I^§^	NT	Yes	I/II	[90–92]

NT, not tested; NR, not relevant; NP, not published.

* No structural data available, based on binding mode of a close analogue;

§ No structural data available, based on binding mode derived from computational docking into AuroraA kinase [93];

¶ No structural data available, based on binding mode derived from computational docking into ERK5 kinase domain [66];

† No structural data available, based on binding mode derived from computational docking into a MEK5 homology model [61];

‡ No structural data available, based on binding modes of close analogues to other kinases.

## The challenge of monitoring ERK5 inhibition in cells

The use of protein kinase inhibitors in biological systems requires clear and specific biomarkers that confirm target inhibition and allow interpretation of the experiment. For example, inhibition of the RAS–RAF–MEK1/2–ERK1/2 pathway with a MEK1/2 or ERK1/2 inhibitor causes loss of phosphorylated ERK1/2 and/or RSK (direct substrates of MEK1/2 and ERK1/2, respectively). Monitoring the ERK1/2 pathway is made easier by the existence of more than 200 ERK1/2 substrates and interacting proteins [41].

Whilst inhibition of MEK5 is relatively easily assessed in cells by loss of ERK5 activation-loop TEY phosphorylation, the paucity of well-validated ERK5 substrates allowing reliable monitoring of ERK5 activity or inhibition has held back our understanding of ERK5 biology, and ERK5i development and disease positioning. Techniques that have been used to monitor ERK5i-dependent inhibition of ERK5 in cells include:

**1. KiNativ™**. This technique measures binding of small molecules to the kinase active site. It uses biotin-tagged acyl-phosphates of ATP and ADP as probes, which acylate the conserved active site lysines in protein kinases (and other ATP-dependent enzymes). Mass spectrometry is then used to identify streptavidin captured peptides following tryptic digestion. Pre-treatment of cells with and without inhibitors, followed by cell lysis and capture of non-inhibitor-bound ERK5 via the ATP-site probe allows the quantification of target engagement [42,43]. This technique does not measure kinase or transcriptional activity.

**2. ERK5 autophosphorylation assay**. MEK5-catalysed phosphorylation of the activation-loop TEY motif activates the ERK5 kinase domain, which subsequently drives autophosphorylation at multiple sites within the C-terminus (Figures 1 and 2). This multi-site phosphorylation causes a pronounced reduction in mobility of ERK5 on SDS–PAGE gels [44,45]. Since this ERK5 ‘band shift’ reflects kinase domain-catalysed autophosphorylation, its loss in cells treated with an ERK5i reports inhibition of ERK5 (Figure 1A). This assay is straightforward as it simply requires a reliable ERK5 antibody. However, at least some phosphorylation sites in the C-terminus are targeted in *trans* by other protein kinases. Furthermore, this assay does not take into account the function of the C-terminus and does not measure the activity of the ERK5 TAD.

**3. ERK5-driven AP-1 reporter assay**. The AP-1 transcription factor consists of homo- and heterodimers of the bZIP transcription factors JUN (JUN, JUNB and JUND) and FOS (FOS, FOSB, FRA1 and FRA2) which bind to the core DNA sequence 5′-TGAG/CTCA-3′. A multimerised AP-1:Luc reporter can be stimulated by ERK5 when it is activated by co-transfected MEK5D (a constitutively active MEK5 mutant). This has been used to assess ERK5 activation and inhibition by ERK5i in cells [44]. However, the sheer complexity of AP-1 means it is very difficult to understand and interpret what is being measured in an ERK5-driven AP-1:Luc reporter assay. AP-1 activity is determined by dimer composition, the abundance of dimer partners (a function of *de novo* expression and proteasomal degradation) and their activity (controlled through phosphorylation, acetylation and protein:protein interactions). In terms of dimer composition, some FOS or JUN proteins can also heterodimerise with the ATF transcription factors (ATF2, ATF3 and B-ATF), MAFs (MAFB, MAFF, MAFG and MAFK) and the cAMP response element-binding proteins (CREBs). In terms of abundance and activity, the ERK1/2 [46,47,48] and JNK [49] pathways drive the expression and phosphorylation of multiple AP-1 proteins, increasing their abundance and activity. ERK1/2 signalling is a positive regulator of the FOS proteins, and ERK1/2 also phosphorylates JUNB and JUND; JNK phosphorylates and activates JUN and ATF2 [46,49].

The most likely mechanism by which ERK5 might regulate AP-1 is by promoting JUN expression. JUN expression is decreased in ERK5^−/−^ MEFs and this is rescued by re-expression of ERK5 [50]. The *JUN* promoter is regulated by MEF2C, the best characterised substrate of ERK5; ERK5 phosphorylation of MEF2C enhances its transcriptional activity and promotes JUN expression [17,51]. ERK5 can also stimulate the phosphorylation and activation of CREB [52] and can phosphorylate ATF2 *in vitro* [20], however, ERK5 does not phosphorylate the Maf family member, MafA [53]. Taking these studies into account, ERK5-dependent regulation of AP-1:Luc is indirect, complex and mostly likely through an ERK5 → MEF2C → JUN pathway (Figure 1B), which is further complicated by other pathway inputs.

**4. MEF2-dependent reporter assays**. The best validated ERK5 interacting proteins and substrates are the MEF2A, C and D transcription factors [10,17,54]. Thus, the MEF2 proteins remain the most appropriate direct reporters of ERK5 activity and have been used in cellular reporter systems for monitoring MEK5 and/or ERK5 inhibition [55–57,58]. The simplest version consists of multimerised MEF2 binding sites and basic promoter elements to drive the expression of a reporter (typically firefly luciferase) in response to EGF (Figure 1B top) [57], making it a significant advance in ERK5 selectivity over AP-1. A potential disadvantage of this assay is that it may ‘report’ the activity of any one of the MEF2 proteins that can bind to the minimal binding site and it may also report on the activity of MEF2-interacting proteins that may be regulated by other pathways. This assay can be made more specific for ERK5 by driving ERK5 activation with co-transfected MEK5D.

A variation of this assay involves fusing the region of MEF2 housing ERK5 phosphorylation sites and the MEF2 TAD to the DNA-binding domain of the yeast transcription factor GAL4 (Figure 1C). This MEF2-GAL4 fusion can then be expressed together with a luciferase reporter controlled by multimerised GAL4 response elements (GREs), ERK5 and MEK5D to stimulate the pathway. This system has been shown to work for MEF2A, C and D [17] and although it has more components, it is a far more direct readout of ERK5 activation since ERK5 interacts directly with the MEF2-GAL4 reporter construct to drive luciferase expression. We have used this assay to measure the pharmacological inhibition of MEK5 and ERK5 [38,55,56].

Transcription-based readouts of ERK5 activity do not readily differentiate between the contribution of the kinase and TAD activities when a full length construct of (or endogenous) ERK5 is assessed. For example, we have found that a truncation mutant of ERK5 that lacked the TAD could drive MEF2D:GAL4 activity when co-transfected with MEK5D, but also that a kinase dead full-length form of ERK5 (that contains the TAD) could drive MEF2D:GAL4 luciferase activity following ERK5i treatment (see below) [55]. However, by using these different constructs, the contribution of the kinase domain and TAD can be delineated.

## Development of MEK5 and ERK5 kinase inhibitors

**Figure 3.**
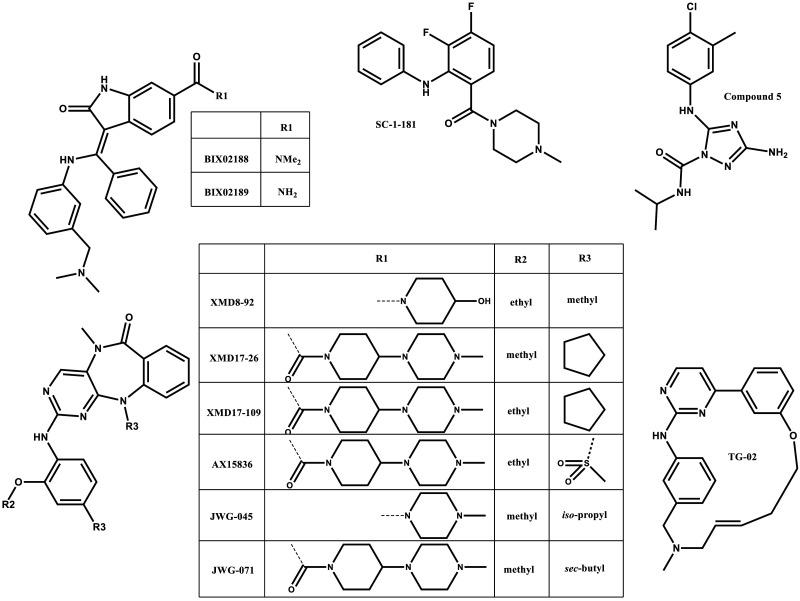

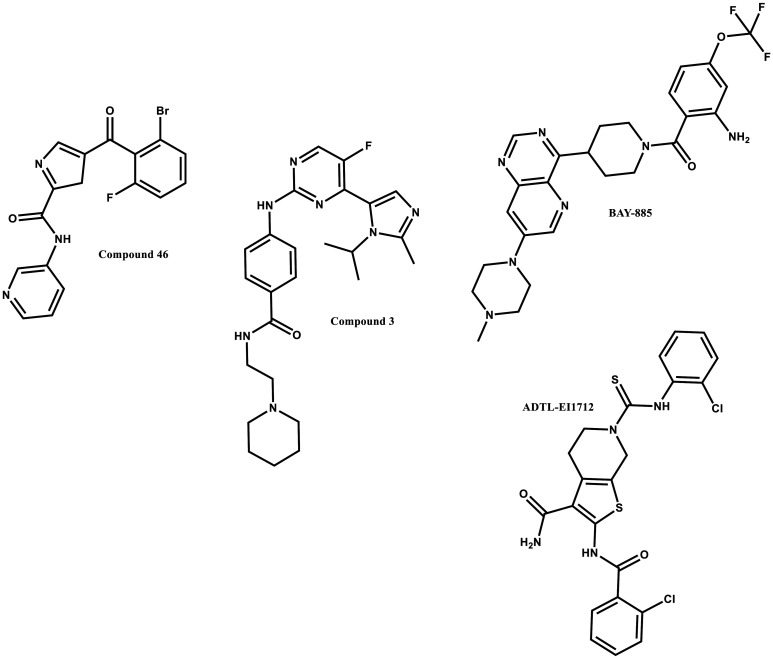
ERK5 inhibitors.

By analogy with the RAF–MEK1/2–ERK1/2 pathway, one could conceive of targeting the ERK5 pathway at any step from MEKK2/3 to MEK5 or ERK5 itself (Figure 1). However, unlike RAF, which appears to be very selective for activation of MEK1/2, MEKK2/3 are able to activate other MKKs in addition to MEK5 [59] such that inhibition of MEKK2/3 may impact on JNK, p38 or ERK1/2 activity as well as ERK5. This may explain why efforts to selectively target ERK5 signalling have focused on MEK5 or ERK5. A summary of current MEK5 and ERK5 inhibitors is presented in Table 1 and Figure 3.

The first pathway inhibitors to be reported were the MEK5 inhibitors, BIX02188 and BIX02189. These inhibitors are relatively selective for MEK5, with the strongest off-target effect on Src [58]. They have been useful for dissecting the role of the MEK5–ERK5 pathway in cells [28,60]. More recently, SC-1-181 has been described; however, the selectivity data have yet to be reported [61,62].

The majority of effort has focused on the development of small molecule ERK5i, probably because ERK5 is the ‘effector kinase’ in the pathway. However, ERK5 is proving to be a challenging target. The first ERK5i to be described were XMD8-92 [63], cpd **25** (XMD17-26) and cpd **26** (also known as XMD17-109 and ERK5-IN-1) [44]. Although selective for ERK5 over other kinases, they had off-target effects on the bromo-domain containing protein, BRD4 [64] — an epigenetic reader involved in transcriptional regulation [65]. AX15836, an analogue of XMD8-92, was engineered to lack bromo-domain activity and is a potent and selective inhibitor of ERK5 kinase activity. However, cell-based experiments showed that AX15836 did not phenocopy genetic ERK5 knockdown. This brings into question results obtained using XMD8-92. Even though XMD8-92 phenocopies the biological effects of siRNA knockdown of ERK5, it is likely this arises through different mechanisms; XMD8-92, through binding to bromo-domain containing proteins, and siRNA to ERK5 by ablating the kinase domain and the C-terminus (including the TAD). This study also suggested that the ERK5 C-terminal domain is important for the biological function of ERK5 [64]. Heedful researchers have since used BRD4 inhibitors, such as JQ1, to delineate the role of BRD4 in their systems [25,45,55,56] and testing of ERK5 inhibitors against BRD4 is now an essential step in ERK5 drug discovery. Adding to the complexity, we have found that cpd **26** (XMD17-109) and AX15836, cause a conformational change in the kinase domain which leads to exposure of the C-terminal NLS and paradoxical activation of the ERK5 TAD [55] (see below and Figure 2).

**Figure 4.**
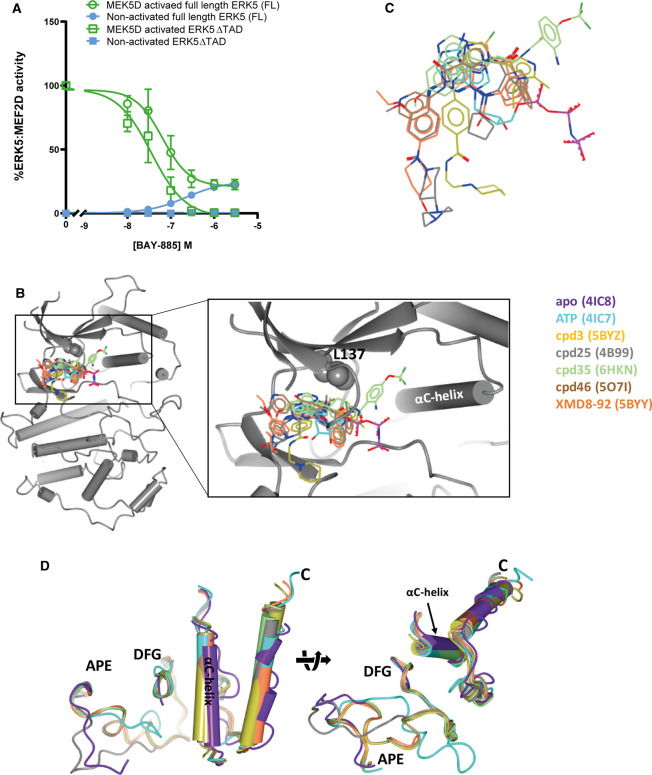
ERK5 small molecule inhibitor binding to the ERK5 kinase domain. (**A**) BAY-885 induces transcriptional activity in the ERK5:MEF2D reporter system. HEK293 cells were transfected with GAL4-MEF2D, GAL4:LUC and CMV:Renilla, together with either wild-type HA-ERK5 (full length) or HA-ERK5ΔTAD and EGFP-MEK5D or EGFP (control) as indicated. Four hours post-transfection, cells were treated with either DMSO (control) or BAY-885. Twenty-four hours post transfection, cells were lysed and firefly luciferase activity was measured and normalised to Renilla. The results are presented as the mean of three independent experiments ± SEM. For full method see [55]. (**B**,**C**) Comparison of the binding mode of selected ERK5 inhibitors with ATP highlights the divergence in solvent channel groups and the extension of the BAY series (exemplified by cpd35) toward the back pocket. (**B**) Inhibitors shown within the context of ERK5 (cartoon representation); the gatekeeper residue (L137) is shown as spheres and the αC-helix is labelled. (**C**) ATP-binding site viewed from the N-terminal lobe of ERK5; protein atoms omitted for clarity. (**D**) Comparison of the conformations of the ERK5 activation loop (N-terminal DFG motif to C-terminal APE motif), αC- and C-terminal helices in the presence and absence of ATP and selected inhibitors demonstrates the similarity in positioning of the αC- and C-terminal helices in the inhibitor- and ATP-bound structures and their divergence from the apo structure, whilst the majority of inhibitor-bound activation loops adopt a similar conformation, they all differ from both the apo and ATP-bound states. In panels (**B**–**D**), inhibitor carbon atoms (**B**,**C**) or protein cartoon (**D**) are coloured as indicated in the key. Crystal structures of ERK5 kinase domain alone and in complex with ATP or selected ATP-competitive inhibitors were overlaid using the ssm algorithm in CCP4MG [94]. Co-ordinates were extracted from the PDBe [https://www.ebi.ac.uk/pdbe/] using the entry codes indicated in the key.

Additional ERK5i in the public domain include JWG-045 and JWG-071, a further development of the XMD8-92 series. Both have selectivity over BRD4 but inhibit LRRK2 and are classed as dual ERK5/LRRK2 inhibitors. The kinase selectivity data for JWG-071 have been published, showing that it only inhibits three other kinases (LRRK2, PLK2, DCAMKL1 and 2) at low micro molar concentrations. In cell-based assays, an inhibitor that blocks LRRK2 activity (such as JWG-048 [45]), and not ERK5 activity, could be used to confirm ERK5 kinase involvement. A multi-site collaboration reported cpd **46**; this inhibitor is distinct from the XMD8-92 series, has no activity towards BRD4 or LRRK2, is highly selective for ERK5 and is also suitable for use in animal studies [56]. Bayer have reported BAY-885, which like AX15836 is very selective for ERK5 and did not significantly inhibit other kinases or BRD4 [57]. However, like AX15386 it also paradoxically activates ERK5 transcriptional activity (Figure 4A and Table 1).

Recently a triple ERK1/2/5 inhibitor, ADTL-EI1712, has been reported [66]. This was developed to simultaneously block the ERK1/2 and ERK5 pathways in cancers where ERK5 activity can compensate for inhibition of the RAS–RAF–MEK1/2–ERK1/2 pathway [31,34]. Two other kinases, Kit and Axl are also inhibited to a similar extent as ERK1, ERK2 and ERK5, but BRD4 engagement was not assessed [66].

All the above inhibitors are type I inhibitors, and have either been shown experimentally or predicted to bind to the active form of ERK5 where the phenylalanine from the activation-loop DFG motif is ‘in’, the catalytic αC-helix is ‘in’, the inhibitors occupy part of the adenine binding pocket and form hydrogen bonds with the kinase hinge region (which connects the N and C-terminal lobes of the enzyme) (Figure 4B). For a review on the classification of kinase inhibitors see [67]. A type IV ERK5 inhibitor, cpd **5**, has been identified by Chen et al. [68]. Type IV inhibitors do not bind the ATP or peptide substrate binding sites and are considered to act as allosteric inhibitors. Although cpd **5** does not bind directly to the ATP-binding site, it displaces the kinase P-loop into the ATP-binding site and thus is ATP competitive. This inhibitor has off-target effects against five other kinases at concentrations required to inhibit ERK5 in cells [68] but it has not been tested against BRD4.

Some ERK5i have anti-tumour activity (Table 1). As XMD8-92, ADTL-EI1712 and TG-02 all have off-target effects (either on BRD4 or other kinases), it is impossible to conclude whether or not this is a direct consequence of ERK5 kinase inhibition (even when it phenocopies knockout techniques, for the reasons discussed above). Cpd **46** is a selective ERK5i, with mild paradoxical activation effects, that prevents tumour growth by inhibiting angiogenesis [56]. These findings show that it is important to understand how targeting ERK5 works — inhibition of tumour cell proliferation or survival, inhibition of tumour angiogenesis, or in appropriate models, reducing the tumour promoting role of the immune system.

## Paradoxical signalling induced by ERK5 kinase inhibitors differs from other kinases — classification of kinase inhibitor-induced paradoxical activation

The unintended activation of kinase signalling through inhibitor binding to the kinase, termed paradoxical activation, has emerged as a significant challenge to kinase inhibitor development. The first kinase that was reported to be paradoxically activated by selective small molecule inhibitors was PKB. Inhibitor binding promotes PKB membrane localisation, regulatory site phosphorylation (T308 and S473) by PDK1 and mTORC2, and acquisition of a phosphatase-resistant conformation; consequently, when the inhibitor is removed the ‘primed’ kinase is fully active [69,6]. Other kinases that undergo ‘priming paradoxical activation’ (Figure 2) include PKC [70], PKD [71], AMPK [72] and JAK2 [73,74,75]. This mechanism can have severe physiological consequences; for example, a life-threatening cytokine-rebound syndrome occurs when the JAK2 inhibitor, ruxolitinib, is withdrawn too quickly and this is due to priming paradoxical activation of JAK2 [73,74,75].

The second and best-known example of paradoxical kinase activation is seen with the first generation BRAF^V600E/K^ inhibitors vemurafenib and dabrafenib. These inhibitors were developed to selectively inhibit BRAF^V600E/K^ and although they are effective in treating BRAF^V600E/K^-driven melanoma they cause adventitious tumour progression in non-melanoma tissue. This is because inhibitor binding to wild-type RAF isoforms induces RAS-GTP-dependent CRAF homodimers or BRAF-CRAF heterodimers in which the drug-bound protomer *trans*-activates the drug-free promoter leading to MEK1/2–ERK1/2 activation [76,77] (Figure 2). Another example of this ‘paradoxical activation by trans-activation’ is bosutinib binding to the pseudokinase HER3 which induces an EGFR-dependent proliferative signal [78,79] (Figure 2). These examples highlight the importance of anticipating whether a kinase is susceptible to inhibitor-induced paradoxical activation.

We have recently shown that the binding of cpd **25** (XMD17-26), **26** (XMD17-109) or AX15836 to the ERK5 kinase domain promotes NLS and TAD exposure which in turn promotes nuclear localisation and paradoxical activation of ERK5 transcriptional activity [55]. The ERK5 kinase domain and the NLS and TAD normally inhibit each other through an intramolecular interaction that is relieved by ERK5i binding (Figure 2). Generation of inhibitor-resistant ERK5 mutants confirmed that this was an ‘on target’ effect of ERK5i binding to the kinase domain. This represents a new mechanism redolent of steroid hormone receptor activation, where ligand binding induces nuclear translocation and transcriptional activity. Thus, we have termed this ‘auxiliary domain paradoxical activation’ (Figure 2).

Do all ERK5i induce paradoxical activation? We have subsequently tested XMD8-92, cpd **46** and BAY-885 in the MEF2D:GAL4 assay (Figure 1). All three paradoxically activate ERK5 in the assay, albeit to differing degrees (Figure 4A, Table 1). We have compared the binding of XMD8-92, cpd **25** (XMD17-26), cpd **35** (an analogue of BAY-885) and cpd **46** to ERK5 to ATP (Figure 4B,C). These ERK5i all occupy a similar space within the adenosine-binding region of the ATP-binding site (Figure 4B,C) and engage in hydrogen bonds with the hinge region, whilst they differ somewhat in the positioning of their solvent-exposed moieties. In addition, cpd **35** extends to occupy an area termed the ‘back pocket’. This region, which lies between the ‘gatekeeper’ residue (L137 in ERK5) and the αC helix, is not occupied by ATP. Comparison of the conformation of the ERK5 αC helix and activation loop in the presence and absence of inhibitors or ATP (Figure 4D) reveals that the inhibitors bind to a more ‘active-like’ conformation of ERK5. Furthermore, our structural simulations with AX15836 showed a reduction in flexibility of the activation loop [55]. The exact mechanism that causes NLS exposure and TAD paradoxical activation when ERK5i bind to the ERK5 kinase domain remains elusive. It could be conformational changes elicited by inhibitor binding, solvent-exposed moieties preventing auto-inhibition, effects on Hsp90 binding, post-translational modifications or a combination of events. We ruled out changes in phosphorylation of known key sites on ERK5 [55], but ERK5 SUMOylation remains to be tested.

Promoting nuclear localisation of ERK5 is an undesirable consequence of ERK5i. Nuclear ERK5 is linked to a high Gleason grade, bone metastasis and ultimately a poor prognosis in prostate cancer [35,80–82]. In HCC there is an increase in ERK5 nuclear localisation but no increase in ERK5 kinase activity [83], and overexpression of the oncogene, Cdc37, promotes kinase-inactive nuclear localisation of ERK5 while increasing cell proliferation [22]. Thus, ERK5i that promote nuclear localisation in tumours with predominately cytosolic ERK5, may exacerbate tumour progression. If tumours have kinase active nuclear ERK5, ERK5i may have some effect if the kinase activity is driving tumorigenesis, but not if ERK5 transcriptional activity is required. Some tumours already have nuclear kinase-inactive ERK5 [83], here ERK5i may increase its transcriptional activity. This highlights the importance of understanding the mechanism of action of ERK5i and determining whether ERK5 kinase and/or transcriptional activity is required for the cellular role of ERK5.

## Conclusions

These findings show that it is essential that all ERK5i are tested not just for off-target activity against kinases and bromo-domain containing proteins, but also for promotion of nuclear localisation and paradoxical transcriptional activation using appropriately sensitive assays. For ERK5 this can be assessed using the ERK5:MEF2D reporter assays, such as the GAL4-MEF2D driven GRE reporter (Figures 1B,C). It should be noted that when using exogenously expressed ERK5, a large tag on the N-terminus, such as GST or GFP will hide this effect and should be avoided (PAL, unpublished results). It also remains to be seen whether other kinases with auxiliary functional domains are activated by small molecules in a similar way to ERK5. To delineate the role of the ERK5 kinase domain in cells, AX15836, cpd **46** and BAY-885 are ERK5 kinase selective inhibitors, but all cause TAD paradoxical activation (to varying degrees) so care must be taken when interpreting results. This highlights the need to find a selective paradox-breaking ERK5i or to use an alternative approach such as a PROTAC [84] to completely remove ERK5 from cells. Critically, these results also raise the question of the relative importance of the ERK5 kinase domain and NLS/TAD functions in normal ERK5 biology and in diseases where ERK5 is implicated.

## Dedication

We dedicate this article to the memory of Michael Wakelam a dear friend, mentor, colleague, a world leader in lipid signalling and lipidomics, and Director of the Babraham Institute until his death in the 2020 Covid-19 pandemic.

## Perspectives

- *Importance of the field*: Protein kinase inhibitors can induce unintended paradoxical signalling. Paradoxical kinase activation can exacerbate disease, such as stimulating tumour growth. ERK5 nuclear localisation and transcriptional activity is paradoxically stimulated by ERK5i.
- *Current thinking*: Characterising the mode of action of kinase inhibitors on their targets using appropriately sensitive assays is critical for all drug discovery programmes. Determining whether ERK5i promote transcriptional activation of ERK5 is essential for any new potential ERK5 therapeutic.
- *Future directions*: Activation of ERK5 transcriptional activity by ERK5i raises significant questions about the role of ERK5 kinase and transcriptional activities in ERK5 biology, including disease biology and therapeutics.

## Open Access

Open access for this article was enabled by the participation of University of York in an all-inclusive *Read & Publish* pilot with Portland Press and the Biochemical Society under a transformative agreement with JISC.

## Abbreviations

ABL: Abelson murine leukaemia homolog
AMPK: 5′ AMP-activated protein kinase
AP-1: activator protein-1
ATF: activating transcription family
AXL: AXL, coming from the Greek word ‘anexelekto’, means uncontrolled
BRD4: bromo-domain-containing protein 4, a transcriptional and epigenetic regulator
bZIP: basic leucine zipper domain
CDC37: cell division cycle 37
CDK1/2/3/4/5/7/9: cyclin dependent kinase1/2/3/4/5/7/9
CREB: cAMP response element-binding protein
CSF1R(FMS): colony-stimulating factor 1 receptor (Feline McDonough Sarcoma)
DCAMKL1/2: doublecortin and CaMK (Ca^2+^/calmodulin-dependent protein kinase)-like 1/2/3
EGF: epidermal growth factor
EGFR: epidermal growth factor receptor
ERK1/2: extracellular signal-regulated protein kinase 1/2
ERK5: extracellular signal-regulated protein kinase 5
ERK5i: small molecule ERK5 inhibitor
FGFR1: fibroblast growth factor receptor 1
FLT3/4: FMS (Feline McDonough Sarcoma)-like tyrosine kinase 3/4
FOS: cellular homolog of the viral oncoprotein v-fos
FRA: Fos-related antigen
Fyn: src family kinase
GAL4: yeast transcription factor
GSK3α: glycogen synthase kinase 3α
HER3: human epidermal growth factor receptor 3
Hsp90: heat-shock protein 90
JAK1/2: Janus kinase 1/2
JNK: c-Jun N-terminal kinases
JUN: cellular homolog of the viral oncoprotein v-jun
LCK: lymphocyte-specific protein tyrosine kinase
LRRK2: leucine-rich repeat serine/threonine-protein kinase 2
MAF: cellular homolog of the viral oncoprotein v-maf (musculoaponeurotic fibrosarcoma)
MAPK: mitogen-activated protein kinase
MAP3K15: mitogen-activated protein kinase kinase kinase 15
MEF2 A, C and D: myocyte enhancer factor-2 A, C and D
MEKK2/3: MAPK/extracellular signal-regulated kinase kinase 2/3
MEK5: MAPK kinase 5
MEK5D: constitutively active form of MEK5 where the regulatory activation-loop phosphorylation sites are mutated to aspartic acid to mimic phosphorylation
MKK: MAP kinase kinase
MKKK: MAP kinase kinase kinase
MST2: mammalian serine/threonine 2 kinase
mTORC2: mammalian target of rapamycin complex 2
NLS: nuclear localisation signal
P38δ: class of mitogen-activated protein kinases that are responsive to stress stimuli
PDK1: phosphoinositide-dependent kinase-1
PH: pleckstrin homology
PKC: protein kinase C
PKD: protein kinase D
PROTAC: proteolysis targeting chimeras
RAF: rapidly accelerated in fibrosarcoma kinase
RAS: Rat sarcoma small GTPase
RBD: RAS binding domain
RSK2/4: ribosomal protein S6 kinase 2/4
SLK: Ste20-like serine/threonine protein kinase
Src: short for sarcoma kinase
STK: spleen tyrosine kinase
TAD: *trans*-activation domain
TYK2: non-receptor tyrosine-protein kinase 2
TYRO3: tyrosine-protein kinase receptor
